# A comprehensive comparison of four species of Onchidiidae provides insights on the morphological and molecular adaptations of invertebrates from shallow seas to wetlands

**DOI:** 10.1371/journal.pone.0196252

**Published:** 2018-04-26

**Authors:** Guolv Xu, Tiezhu Yang, Dongfeng Wang, Jie Li, Xin Liu, Xin Wu, Heding Shen

**Affiliations:** 1 National Demonstration Center for Experimental Fisheries Science Education (Shanghai Ocean University), Shanghai, China; 2 International Research Center for Marine Biosciences at Shanghai Ocean University, Ministry of Science and Technology, Shanghai, China; 3 Key Laboratory of Exploration and Utilization of Aquatic Genetic Resources (Shanghai Ocean University), Ministry of Education, Shanghai, China; Zhejiang University College of Life Sciences, CHINA

## Abstract

The Onchidiidae family is ideal for studying the evolution of marine invertebrate species from sea to wetland environments. However, comparative studies of Onchidiidae species are rare. A total of 40 samples were collected from four species (10 specimens per onchidiid), and their histological and molecular differences were systematically evaluated to elucidate the morphological foundations underlying the adaptations of these species. A histological analysis was performed to compare the structures of respiratory organs (gill, lung sac, dorsal skin) among onchidiids, and transcriptome sequencing of four representative onchidiids was performed to investigate the molecular mechanisms associated with their respective habitats. Twenty-six SNP markers of *Onchidium reevesii* revealed some DNA polymorphisms determining visible traits. Non-muscle myosin heavy chain II (NMHC II) and myosin heavy chain (MyHC), which play essential roles in amphibian developmental processes, were found to be differentially expressed in different onchidiids and tissues. The species with higher terrestrial ability and increased integrated expression of *Os-MHC* (NMHC II gene) and the MyHC gene, illustrating that the expression levels of these genes were associated with the evolutionary degree. This study provides a comprehensive analysis of the adaptions of a diverse and widespread group of invertebrates, the Onchidiidae. Some onchidiids can breathe well through gills and skin when under seawater, and some can breathe well through lung sacs and skin when in wetlands. A histological comparison of respiratory organs and the relative expression levels of two genes provided insights into the adaptions of onchidiids that allowed their transition from shallow seas to wetlands. This work provides a valuable reference and might encourage further study.

## Introduction

Environmental adaptations, which arise through natural selection, have both physiological and molecular mechanisms [1–3]. Studies of adaptive traits are important for understanding the evolution of respiration, movement and other features [4,5]. Some aquatic vertebrates, such as mudskippers [5] and lungfish [6], have developed terrestrial adaptations that enable them to spend considerable amounts of time on land. However, few systematic studies have attempted to identify the mechanisms of adaptive evolution in invertebrates, and the molecular and morphological bases of adaptive evolution in invertebrates remain largely unknown.

**Fig 1:**
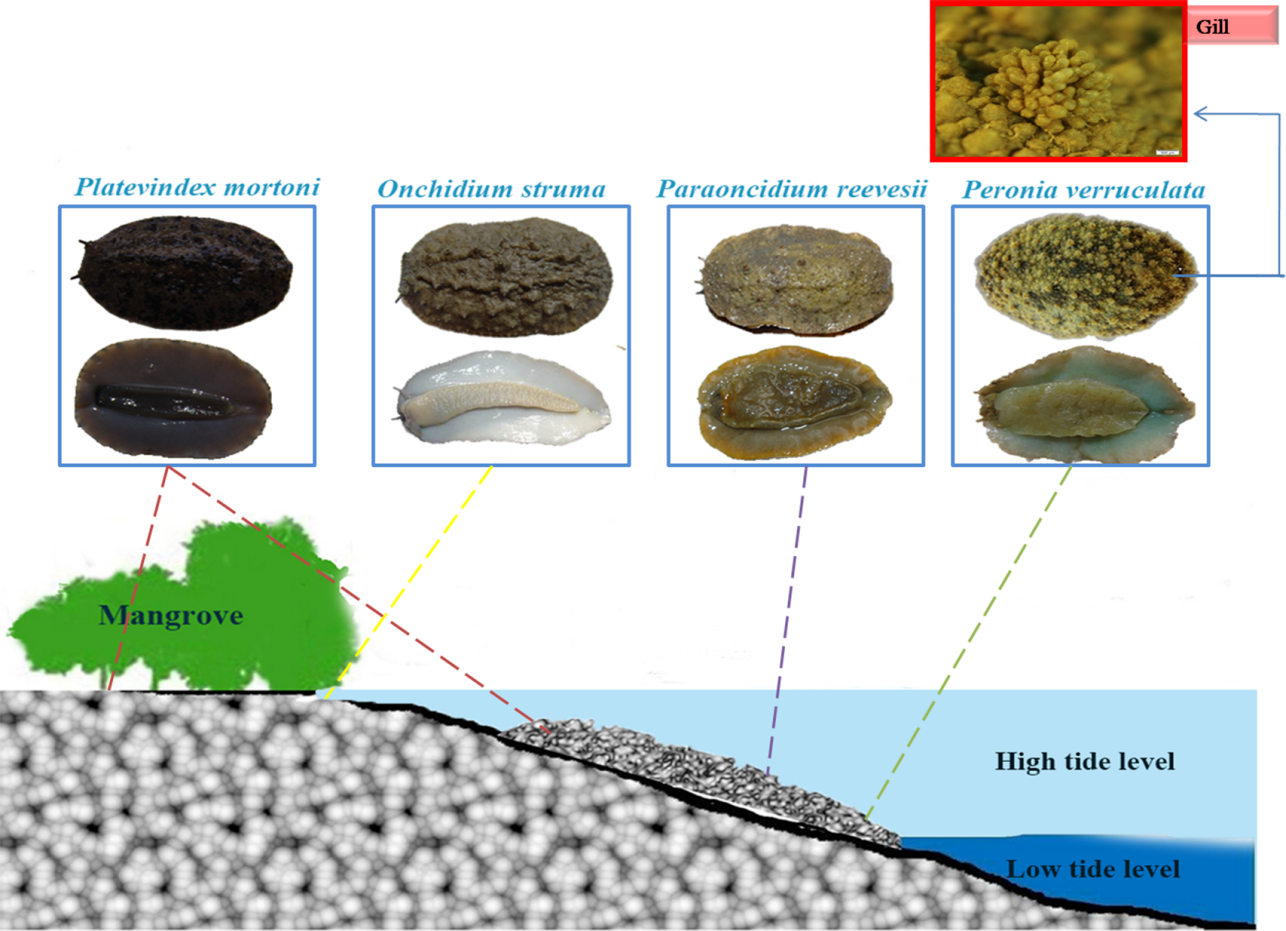
Habitats of the four studied species from the family Onchidiidae. The illustration was created using Photoshop. *Onchidium reevesii* primarily occupies wetlands, *Platevindex mortoni* can survive well in both water and wetlands, and *Paraoncidium reevesi*i and *Peronia verruculata* predominantly dwell in water. Note: The photograph outlined in red shows the dendritic gills in the dorsal skin of *Peronia verruculata*.

The family Onchidiidae (Gastropoda: Eupulmonata: Onchidioidea) provides ideal invertebrate models for studying amphibious adaptations because it includes few taxonomic groups composed of both aquatic-living organisms and primarily terrestrial-living pulmonate organisms. The family Onchidiidae, which belongs to a clade of eupulmonates, is mainly composed of marine intertidal, shell-less, air-breathing slugs. With the exception of the family Ellobiidae, Onchidiidae is the only family of Eupulmonata with a free-life veliger stage [7]. Onchidiidae species are widely distributed in the intertidal zone of the South China Sea, the East China Sea and South Yellow Sea and in estuarine mangrove areas [8]. Six species in five genera have been identified in China [9], and the following four of these are widely distributed in China: *Peronia verruculata*, *Paraoncidium reevesii*, *Onchidium reevesii* and *Platevindex mortoni*. Their habitats include shallow seawater, the intertidal zone and the supratidal zone, and these follow a gradual distribution from sea to wetland. *O*. *reevesii* [10], which mainly lives in wetlands, cannot remain under water for long periods; *P*. *reevesii*, which is mainly aquatic, can remain submerged in seawater for long periods and feed on surface algae on coral reefs; and *P*. *mortoni* can live in both shallow seawater and wetlands and has the ability to burrow in mud and climb on rocks. As the only species with dendritic gills that function as a respiratory organ when submerged, *P*. *verruculata* is predominantly an aquatic organism (Fig 1).

Members of Onchidiidae have three types of respiratory organs: dendritic gills, lung sacs and skin. Veligers, which are mainly aquatic, use gills to breathe, and following metamorphosis, these gills are eventually replaced with “lung sacs” as an adaptation to a wetland habitat. A few species continue to use dendritic gills after metamorphosis, and their respiratory methods are similar those of the subclass Opisthobranchia [11]. The different habitats have led to the evolution of different breathing methods [3,12]. Therefore, Onchidiidae is a useful group for understanding the morphological and molecular mechanisms underlying the terrestrial adaptations of amphibious invertebrates.

Multiple species can be studied to represent a continuum of adaptions from more to less terrestrial. However, very little is known about the genetic and histological bases of the different adaptations of onchidiid species. Here, we compared the tissue morphology among four representative species: *Peronia verruculata*, *Paraoncidium reevesii*, *Onchidium reevesii* and *Platevindex mortoni*.

We also conducted *de novo* transcriptome sequencing of the four species. Next-generation sequencing technology makes it feasible and convenient to analyze the transcriptomes of non-model organisms and provides large-scale sequence data. These data are valuable for understanding biological processes, such as metabolic processes and signal transduction [13]. Moreover, to improve our understanding of the population structure of *O*. *reevesii* and differences in epidermis morphology, muscle formation, blood vessel development and cuticularization between this species and others, single nucleotide polymorphism loci were developed and characterized. Genes related to environmental adaption were identified based on our transcriptome data and select SNP loci. Among these single nucleotide polymorphisms (SNPs), we selected the *myosin heavy chain* gene for further analysis. The myosin heavy chain is a tissue-specific protein as well as the primary protein in muscle [14]. In fact, the myosin heavy chain protein is related to the contraction of muscle and is thus of interest when analyzing muscle adaptations. Furthermore, since the discovery of myosins in non-muscle cells, it has been suggested that these proteins drive morphogenesis for successful development [15,16]. Non-muscle myosin II (NM II) is a suitable candidate for analyzing adaptions because it is present in all tissues and is related to morphological development [16]. Non-muscle myosin heavy chain II (NMHC II) is produced from non-muscle myosin II, which is composed of a pair of heavy chains and two pairs of light chains [17]. Therefore, these two genes are suitable candidates for comparing adaptations among the four species of interest. Interestingly, onchidiids express trait-associated genes in various tissues according to their specific habitats. The comparative analyses performed in this study provide insights into the seawater-to-land transition that has occurred in Onchidiidae.

## Materials and methods

### Ethics statement

This study was carried out in strict accordance with the Guidelines on the Care and Use of Laboratory Animals issued by the Chinese Council on Animals Research and Guidelines of animal Care. The study was approved by the ethical committee of Shanghai Ocean University.

### Sample collection

The adult individuals used in this study were collected between May and November. All the animals used in the transcriptome sequencing and qRT-PCR experiments were collected in August and were maintained at 27 ± 1°C for one week prior to the study. *Onchidium reevesii* individuals were collected from Shanghai (31°33′N, 121°48′E); *Paraoncidium reevesii* and *Platevindex mortoni* individuals were collected from Xiamen, Fujian Province (24°27′N, 118°04′E); and *Peronia verruculata* individuals were collected from Zhanjiang, Guangdong Province (21°11′N, 110°24′E). All the individuals of the four species (10 specimens per onchidiid) were fed corn flour and reared at room temperature.

### Stereomicroscopy, light microscopy and scanning electron microscopy

Three fresh adult specimens of each species were anaesthetized by ether, and their external morphologies were observed under a Olympus SZX16 stereomicroscope. Dorsal and ventral skin samples from the four species of Onchidiidae were dissected into small pieces, fixed in Bouin solution and embedded in paraffin wax [18]. Sections (5~6 μm) were cut on a Leica RM2035 microtome, stained with hematoxylin-eosin and observed under a Nikon Eclipse Ni light microscope.

Ten sections were selected randomly from each specimen for measurements of the skin, epidermis, dermis, stratum compactum and stratum spongiosum at six sites. The data were analyzed using the software package JMP Version 10.0 (SAS Inc., NC, USA).

For scanning electron microscopy (SEM) analysis of the Onchidiidae species, tissues were fixed in a mixture of methanol and glutaraldehyde for one week and then preserved in 75% alcohol. After this procedure, the materials were washed three times in phosphate buffer (pH 7.0) for 15 min each time, cleaned in an ultrasonic water bath for 2~3 min and then dehydrated in a series of increasingly concentrated ethanol solutions (30%, 50%, 70%, 80%, 90%, and 100% ethanol), with 15 min per solution. Finally, the samples were prepared for SEM using critical-point drying, sputter coated with gold using DMX-220 ion-plating equipment and then examined by SEM.

### Transcriptome sequencing and sequence analysis

Five active individuals of similar size from each species were selected randomly, and their mantles were removed and individually placed in labeled, RNase-free, 2-ml EP tubes with RNAlater (Qiagen: 76104). All the samples were sent to Genergy Biotechnology (Shanghai) Co., Ltd., for RNA extraction and sequencing. The cDNA library was sequenced by Genergy Biotechnology Company (Shanghai, China) using an Illumina HiSeq^TM^ 2000 (Illumina, Inc., USA). The raw data were processed to remove nonsense sequences and then assembled using the short-reads assembling program Trinity [19,20].

Functional annotation of the transcriptome was performed using Blast2GO software [21–23]. For annotation, BLASTX alignment (e value<1e-5) between unigenes and protein databases, such as UniProt (www.uniprot.org) and NCBI NR (NCBI non-redundant nucleotide database, (http://www.ncbi.nlm.nih.gov/), was performed, and the best aligning results were used to annotate the protein functions. Unigene annotation provided functional annotations of unigenes and included protein sequence similarity, GO (Gene ontology, http://www.geneontology.org/GO.slims.shtml) [24] functional classification, and KEGG (Kyoto Encyclopedia of Genes and Genomes, http://www.genome.jp/kegg/) pathway analysis [25]. Representative sequences of 18S were obtained from the GenBank database for phylogenetic analysis. A phylogenetic tree was constructed using the Bayesian method with MrBayes version 3.2.4. The bootstrap test was employed based on 10,000 pseudo-replications to assess the reliability of the phylogenetic tree.

### SNP marker development in *Onchidium reevesii*

We investigated unigenes from the transcriptome of *Onchidium reevesii* using SAMtools and calling SNPs [26]. Then, potential SNP loci of *O*. *reevesii* that differed from those of *Peronia verruculata*, *Paraoncidium reevesii* and *Platevindex mortoni* related to vascularization, muscle development, cuticularization and epidermis formation were selected. Primer pairs were designed using Primer Premier 5.0 (http://www.premierbiosoft.com) and synthetized by Map Biotech (Shanghai China). The primer pairs were then tested in 10 individuals as a preliminary screen. The primers that produced clearly defined bands were further tested for the analysis of polymorphisms in 60 individuals.

Genomic DNA was extracted from the mantle of living *O*. *reevesii* adults (collected from Chongming Island, Shanghai, China) using a phenol-chloroform extraction method [27]. Multiplex PCR conditions were standardized to a 20-μl volume containing 4 μl of Primer Mix, 1.6 μl of Mg^2+^, 0.4 μl of dNTP Mix, 10 μl of Ex Taq, 2 μl of DNA, and 2 μl of deionized water. The inactivated multiplex PCR product mix was used for SNaPshot multiple single-base extension reactions. The multiple single-base extension reactions were standardized to 25 μl comprising 5 μl of SNaPshot Multiplex Kit (ABI), 2 μl of multiplex PCR product mix, 1 μl of the extension primer mix, and 2 μl of deionized water. The extension reactions were performed under the following conditions: an initial denaturation at 95°C for 10 s followed by 25 cycles of denaturation at 95°C for 10 s, 40 s at 50°C, and 30 s at 60°C and a final extension step at 30°C for 30 s. The products were then tested for polymorphisms using an ABI 3730XL sequencer.

The primary analysis of the ABI 3730XL sequencing data was performed with GeneMapper 4.0 (Applied Biosystems Co., Ltd., USA). The number of alleles (Na), the observed heterozygosity (H_O_), the expected heterozygosity (H_E_) and the deviations from Hardy-Weinberg equilibrium (HWE) for each locus were calculated with Popgene32 (Version 1.32). Bonferroni correction was used to correct the results. The polymorphism information content was calculated with Cervus 3.0 (http://www.fieldgenetics.com/pages/home.jsp).

### Cloning and quantitative analysis of the *Os-NMHC* and *MyHC* genes

Samples of the dorsal skin, ventral skin, foot skin, lung sac, ganglion and ventricle were collected from the four species. The samples (three specimens per tissue) were immediately flash frozen in liquid N_2_ and maintained at -80°C until use. Total RNA was extracted from the tissues with RNAiso Plus (TaKaRa, Japan) according to the manufacturer’s recommended protocol. Briefly, total RNA was obtained from a mixed extraction of tissues. The A260/280 and A260/230 ratios of the RNA were measured using a NanoDrop 2000 spectrophotometer (NanoDrop Technologies, USA) and equaled 1.90–2.10 and 2.00–2.50, respectively. cDNA was synthesized from the dorsal skin mRNA using an RT reagent kit with gDNA Eraser (TaKaRa, Japan), and the 3’ and 5’ ends of the cDNA were obtained using the RACE technique (TaKaRa, Japan).

Partial fragments of the *Os-NMHC* and *MyHC* genes were obtained from the *de novo* transcriptomic library. To confirm the fragment sequences, we used specific primers to amplify the partial fragments and re-sequenced the PCR products. The specific primers used for cloning the full-length cDNA of *Os-NMHC* and *MyHC* are provided in Table 1. The PCR cycling conditions were as follows: 94°C for 5 min followed by 30 cycles of 94°C for 30 s, 58°C for 30 s, and 72°C for 1 min. The smart 5’-RACE (5’ Full RACE Kit, TaKaRa, Japan) and 3’-RACE kits (3’-Full RACE Core Set Ver. 2.0, Takara, Japan) were used per the manufacturers’ instructions. The RACE-PCR product was ligated into pGEM-T Easy vector (Promega, USA) and transformed into competent *Escherichia coli* DH5-α cells. Using blue-white selection and PCR identification, positive clones were selected and sequenced. Concurrently, cDNAs of other tissues were synthesized for qRT-PCR analysis of *Os-NMHC* gene expression. In addition, a constitutively expressed gene, 18S, was used as an internal control to verify the fluorescent real-time RT-PCR reactions.

**Table 1:** PCR primers used in gene cloning.

Usage	Primer name	Primer sequence (5’-3’)	Description
RT-PCR	Test-1F	CCAACCGCACCAGCCGTGAGT	Used to amplify one part of the *Os-NMHC* fragment
	Test-1R	GCGGTCCAGAGATTTGTTGAT	
	Test-2F	TAAGAATAAGTATGAGGCAAT	Used to amplify one part of the *Os-NMHC* fragment
	Test-2R	GCTCCACTGTCATATCGTCCA	
	Test-3F	GACTTCCTACAACTTCGAGCA	Used to amplify one part of the *Os-NMHC* fragment
	Test-3R	CTCTTTCACTCTCTGCTTGTC	
	Test-4F	ACCGCACTAACCCAGGCATTC	Used to amplify one part of the *Os-NMHC* fragment
	Test-4R	CTCTGGATGACACGGATAGCA	
	Test-5F	CTGTATCGCATTGGGCAGAGC	Used to amplify one part of the *Os-NMHC* fragment
	Test-5R	GCTGTGGTGTCCAGGGAATCT	
	Test-6F	AGGAAGAGAACAAGAGAATCAG	Used to amplify one part of the *Os-NMHC* fragment
	Test-6R	AGGAAGAGAACAAGAGAATCAG	
	Test-7F	CCAAGCGTAATGCTGAGTCTG	Used to amplify one part of the *Os-NMHC* fragment
	Test-7R	CATCCTCTTCTCCATCTTTCT	
	Test-8F	TGCGTGGCTATCAACCCC	Used to amplify one part of the *MyHC* fragment
	Test-8R	GCCCTCAAGCACACCGTT	
	Test-9F	AGACTGTGTCCCACTTGC	Used to amplify one part of the *MyHC* fragment
	Test-9R	TGAGCGGACGGATGAGAT	
	Test-10F	GTCAAGAAATACCAGCAG	Used to amplify one part of the *MyHC* fragment
	Test-10R	TAGTGATGATGATGGTGG	
RACE	3’RACE-F1	ATGTCGGATAAAGCCCGCAAAG	Gene-specific outer primer for *Os-NMHC*
	3’RACE-F2	GCACGCACAAAGGCAACC	Gene-specific inner primer for *Os-NMHC*
	3’RACE-F3	GCGGCACACCAAGTTTGACCACAT	Gene-specific outer primer for *MyHC*
	3’RACE-F4	AACGAGGGTGGAATCCGGACTATA	Gene-specific inner primer for *MyHC*
	3’RACE outer primer	TACCGTCGTTCCACTAGTGATTT	Primers from kit
	3’RACE inner primer	CGCGGATCCTCCACTAGTGATTTCACTATAGG	
	5’RACE-R1	TTGGCTTGTAGCAGTTGGTTCTCA	Gene-specific outer primer for *Os-NMHC*
	5’RACE-R2	AAACCCATTGGATTCGTCTG	Gene-specific inner primer for *Os-NMHC*
	5’RACE-R3	GTAGGCATTGTCAGAGAT	Gene-specific outer primer for *MyHC*
	5’RACE-R4	AGGGGTTGATAGCCACGC	Gene-specific inner primer for *MyHC*
	5’RACE outer primer	CATGGCTACATGCTGACAGCCTA	Primers from kit
	5’RACE inner primer	CGCGGATCCACAGCCTACTGATGATCAGTCGATG	
qRT-PCR	qRT-PCR primer F	AGACTGGTCCAAGTATGCCTA	Used to amplify the *Os-NMHC* fragment for real-time PCR
	qRT-PCR primer R	CCATAATGCTCATGGACTCG	
	qRT-PCR primer F	GCCTCCTCATTTGTTCTCCA	Used to amplify the *MyHC* fragment for real-time PCR
	qRT-PCR primer R	ATCTTCTTCTCGGCTCCCTC	
	18S primer F	CGGCTACCACATCCAAGGAA	Used to amplify the 18S fragment for real-time PCR
	18S primer R	GCTGGAATTACCGCGGCT	

Note: We designed seven pairs of primers, and three pairs of primers were used for reverse transcription PCR (RT-PCR) amplification of the coding region, as the lengths of *Os-NMHC* and *MyHC* were too long.

The expression of *Os-NMHC* and *MyHC* transcripts in different tissues was studied by fluorescent real-time RT-PCR. Quantitative RT-PCR was performed using the Light Cycler® 480 II instrument (Roche, Swiss) with a QuantiFast® SYBR® Green PCR kit (Qiagen, Germany). The reaction conditions were as follows: 94°C for 5 min followed by 30 cycles of 94°C for 30 s, 51°C for 30 s, and 72°C for 1 min and a final step at 72°C for 5 min. Data were collected from each qRT-PCR experiment performed in triplicate and expressed as the means ± SE. All the primers used in this process are listed in Table 1.

## Results

### Comparisons of morphological characteristics among the four species

**Fig 2:**
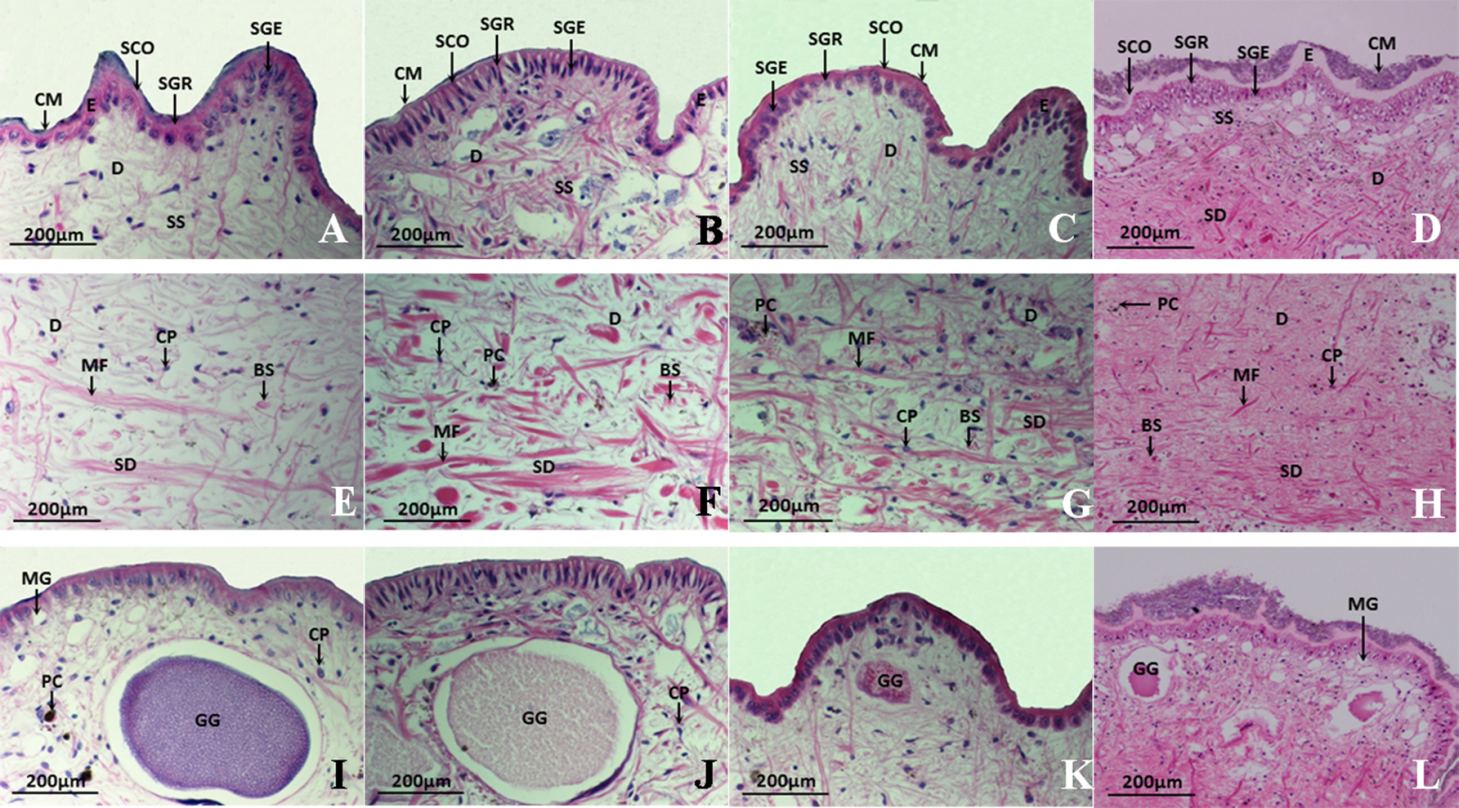
Light microscopy of the dorsal skin of four species in the Onchidiidae family. (**A-D**) An overview of the dorsal skin of (**A**) *Onchidium reevesii* (×40), (**B**) *Paraoncidium reevesii* (×40), (**C**) *Platevindex mortoni* (×40) and (**D**) *Peronia verruculata* (×40). (**E-H**) Dermis layer of (**E**) *O*. *reevesii* (×40), (**F**) *P*. *reevesii* (×40), (**G**) *P*. *mortoni* (×40) and (**H**) *P*. *verruculata* (×40). (**I-L**) Histological observation of glands in four species of Onchidiidae: (**I**) *O*. *reevesii* (×40), (**J**) *P*. *reevesii* (×40), (**K**) *P*. *mortoni* (×40), and (**L**) *P*. *verruculata* (×40). E, epidermis; D, dermis; SS, stratum spongiosum; SC, stratum compactum; CM, cuticular membrane; SCO, stratum corneum; SGR, stratum granulosum; SGE, stratum germinativum; MG, mucous gland; GG, granular gland; PC, pigment cell; MF, muscle fiber; BS, blood sinus; CP, calcium particle.

The stereomicroscopy analysis revealed that the nodular papillae in the dorsal skin were most pronounced in *Onchidium reevesii* among the four species of Onchidiidae. The most prominent difference between *Peronia verruculata* and the other three species was the presence of dendritic gills located at the posterior end of the body. *P*. *verruculata* has these dendritic gills (Fig 1), which allow it to breathe well when submerged. Moreover, the skin over the gills is thin, and thus, the gills have greater permeability than the other parts of the back skin, although they also have thicker cuticular membranes than the gills of the other three species (Fig 2). The surfaces of Onchidiidae species are covered with a layer of cuticular membrane, which becomes purple after staining. The epidermis of Onchidiidae species is composed of two to three cell layers, and the epidermis of *P*. *verruculata* is highly keratinized. The cells of each layer are abundant and closely arranged in *O*. *reevesii* and *Platevindex mortoni*, whereas in *P*. *verruculata* and *Paraoncidium reevesii*, they are sparsely arranged. We measured the dorsal skin thickness of the four species (Table 2) and found that *P*. *verruculata* had the thickest dorsal skin and that *P*. *reevesii* had the thinnest dorsal skin. Granular and mucous glands, which are multicellular glands, were present in the skin of the Onchidiidae species (Fig 2).

**Table 2:** Dorsal skin thicknesses of four species in the family Onchidiidae (Unit: μm).

Species	Epidermis		Stratum spongiosum		Stratum compactum		Whole skin	
	Min ~ Max	Mean ± SE	Min ~ Max	Mean ± SE	Min ~ Max	Mean ± SE	Min ~ Max	Mean ± SE
*Onchidium reevesii*	30.38 ~ 65.08	43.01 ± 5.07**	212.29 ~ 830.61	475.97 ± 103.45**	271.62 ~ 372.50	287.79 ± 20.37	548.20~1156.77	816.74 ± 107.90*
*Paraoncidium reevesii*	20.54 ~ 30.35	26.17 ± 1.89	247.87 ~ 617.63	438.69 ± 67.23	208.16 ~ 364.28	293.30 ± 24.43	531.16 ~ 921.01	764.98 ± 62.65
*Platevindex mortoni*	27.39 ~ 36.02	32.78 ± 1.35*	224.59 ~ 483.42	358.12 ± 35.79	172.55 ~ 425.91	266.36 ± 37.36	473.84 ~ 885.09	662.29 ± 65.58
*Peronia verruculata*	51.77 ~ 110.06	72.06 ± 8.22**	173.19 ~ 409.18	345.05 ± 37.94	371.97 ~ 627.31	486.21 ± 47.24**	846.86 ~ 997.13	914.37 ± 23.1*
*Peronia verruculata*(gill)	26.93 ~ 53.83	37.16 ± 3.99	43.38 ~ 73.39	54.78 ± 4.78**	87.63 ~ 177.73	124.34 ± 14.75**	178.95 ~ 246.05	205.35 ± 12.38**

Statistical analyses of the thicknesses of the epidermis, stratum spongiosum, stratum compactum and whole skin were performed for comparisons among the four species.

* indicates a significant difference (*P*<0.05)

** indicates an extremely significant difference (*P*<0.01).

**Fig 3:**
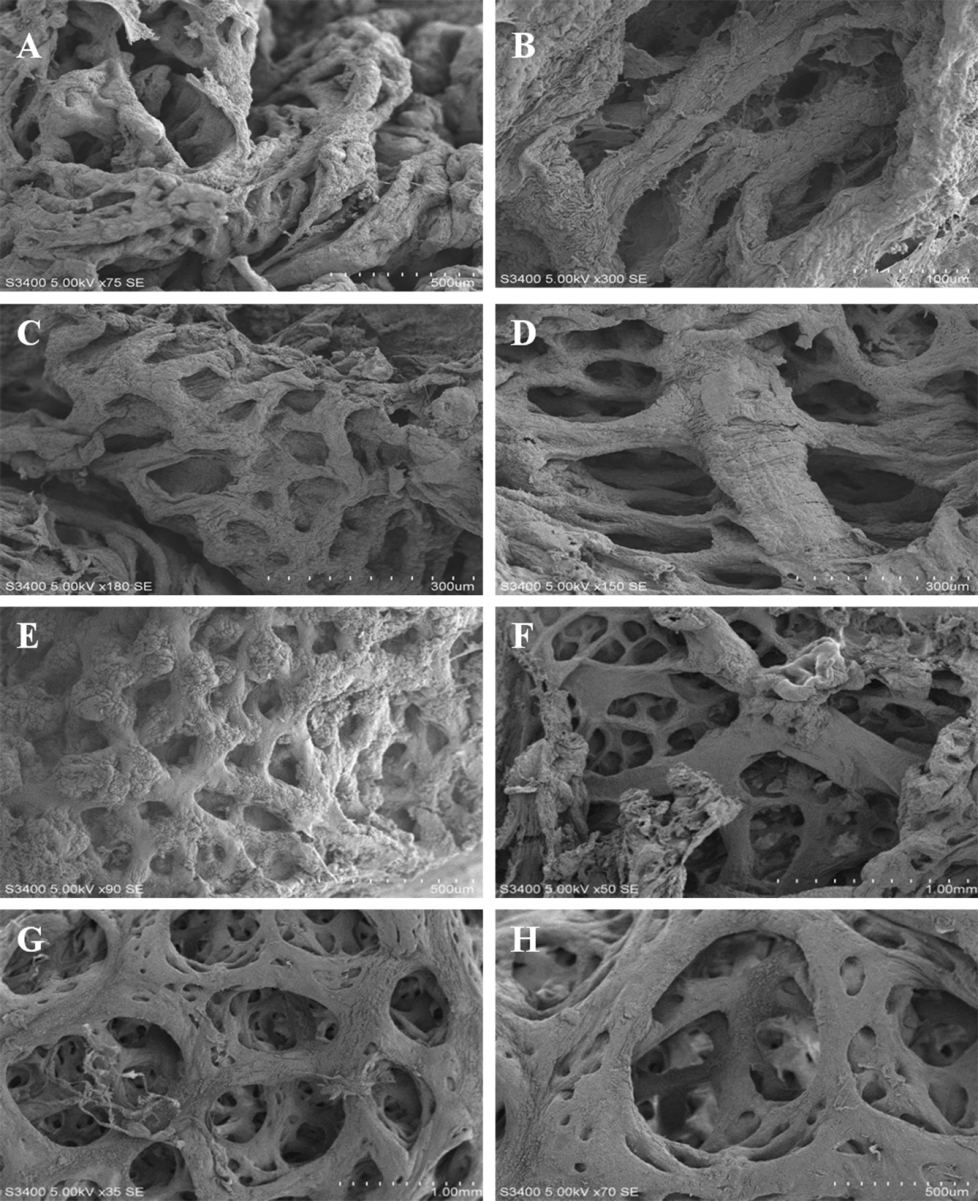
SEM observations of the lung sac of four species in the family Onchidiidae. (A-B). *Paraoncidium reevesii*; (C-D). *Platevindex mortoni*; (E-F). *Peronia verruculata*; (G-H). *Onchidium reevesii*.

*Onchidium reevesii* had the most developed lung sacs, closely followed by *Platevindex mortoni* and *Peronia verruculata*, whereas *Paraoncidium reevesii* had the least-developed lung sacs (Fig 3). The structural differences among the lung sacs of the four species in the Onchidiidae family are described in Table 3. Specifically, *Onchidium reevesii* has developed reticular septa, secondary septa and third septa, whereas *Paraoncidium reevesii* only possesses undeveloped reticular septa (Table 3). The degree of development of the lung sacs in the four species of Onchidiidae decreases in the order *Onchidium reevesii*, *Peronia verruculata*, *Platevindex mortoni* and *Paraoncidium reevesii*.

**Table 3:** Structural differences among the lung sacs of four species in the family Onchidiidae.

	*Paraoncidium reevesii*	*Platevindex mortoni*	*Peronia verruculata*	*Onchidium reevesii*
**Reticular septa**	Small pores, thick walls	Big pores, thick walls	Big pores, thick walls	Big pores, thin walls
**Secondary septa**	None	Developed	Developed	Developed
**Third septa**	None	None	None	Developed
**Diameter of sac rooms (μm)**	0.5–1.5	0.8–5.0	4.5–6.6	5.1–12.7
**Diameter of small room (μm)**	None	0.4–2.7	1.5–4.2	3.4–7.3
**Diameter of subordinate rooms (μm)**	None	None	None	0.7–4.5

### Transcriptome analysis

Four sequencing libraries were constructed from the RNA of the dorsal skin from four species of Onchidiidae. To ensure the high quality of the data for analysis, adaptor sequences, low-quality bases and short reads were removed. After this filtering, we obtained 60,219,324, 89,062,542, 62,624,204, and 61,663,900 reads for *Platevindex mortoni*, *Paraoncidium reevesii*, *Onchidium reevesii* and *Peronia verruculata*, respectively (Table 4). In addition, 131,325 (*Platevindex mortoni*), 233,625 (*Paraoncidium reevesii*), 416,848 (*Onchidium reevesii*) and 263,097 (*Peronia verruculata*) unigenes were annotated successfully by GO annotation. These annotated unigenes were classified into three categories: BP (biological process), CC (cellular compartment) and MF (molecular function) (Table 5).

**Table 4:** Sequence information of four Onchidiidae species.

Sample ID	Sequencing type	Raw read length(bp)	Number of reads	Product size	Effective number of reads	Effective data	Effective rate (%)
**Sample_M**	Pair-End	101	61356624	6197019024 bp (6.197 Gb)	60219324	5841892655 bp (5.842 Gb)	94.2694
**Sample_R**	Pair-End	101	90701864	9160888264 bp (9.161 Gb)	89062542	8617271978 bp (8.617 Gb)	94.0659
**Sample_S**	Pair-End	101	63774300	6441204300 bp (6.441 Gb)	62624204	6073850713 bp (6.074 Gb)	94.29682
**Sample_V**	Pair-End	101	62832016	6346033616 bp (6.346 Gb)	61663900	5987378475 bp (5.987 Gb)	94.34836

**Sample_M,** *Platevindex mortoni***; Sample_R,** *Paraoncidium reevesii*; **Sample_S,** *Onchidium reevesii*; **Sample_V,** *Peronia verruculata*

**Table 5:** Annotated unigenes based on gene ontology.

	*Platevindex mortoni*	*Paraoncidium reevesii*	*Onchidium reevesii*	*Peronia verruculata*
Biological process	68918	124598	221660	137152
Molecular function	29298	51891	90183	60939
Cellular component	33109	57136	105005	65006

**Fig 4:**
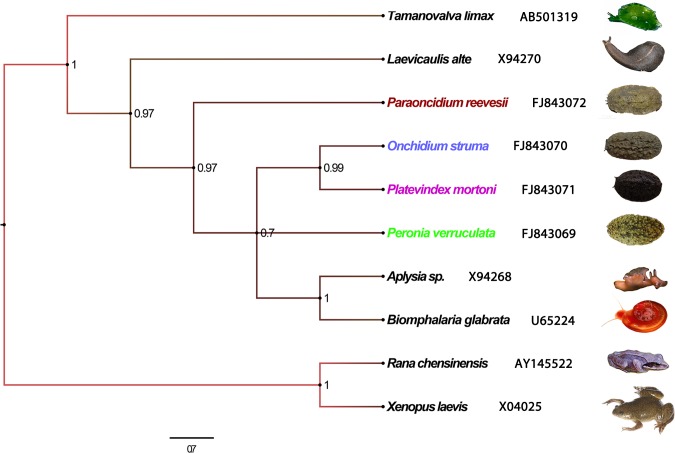
Phylogenetic analysis of 13 species. The names presented in non-black text highlight the main objects of this research study. The phylogenetic tree was inferred using Bayesian methods with MrBayes version 3.2.4. This tree was generated using 18S sequences.

In addition to the GO analysis, a KEGG pathway mapping analysis based on the enzyme commission (EC) numbers, which is an alternative approach for categorizing gene functions with an emphasis on biochemical pathways, was performed using the assembled sequences. This analysis revealed that the detected unigenes participated in 129, 136, 138 and 134 pathways in *Platevindex mortoni*, *Paraoncidium reevesii*, *Onchidium reevesii* and *Peronia verruculata*, respectively. To determine the phylogenetic relationships among the Onchidiidae sequences and their orthologs in other mollusks and amphibians, we constructed a phylogenetic tree using MrBayes version 3.2.4 (Fig 4).

### Development of SNP markers of *Onchidium reevesii*

SNPs are important molecular markers, and the SNPs of Onchidiidae developed in this study were valuable for understanding the species' respiratory traits and amphibious features. The proposed sites in the transcriptome sequences of *Onchidium reevesii* were searched using SAMtools, and 152,212 SNPs were detected after analysis. Fifty-seven alternative SNP loci of *O*. *reevesii* that differed from the SNPs of *Peronia verruculata*, *Paraoncidium reevesii* and *Platevindex mortoni* related to vascularization, muscle development, cuticularization and epidermis formation were selected for further study. Fifteen sequences were selected to design 57 pairs of primers for 57 loci. Forty-two pairs of primers were successfully amplified among these 57 loci, and 26 of these 42 pairs were selected to test for polymorphisms (Table 6). In total, the observed and expected heterozygosities ranged from 0.2553 to 1.0000 and from 0.0000 to 0.7447, respectively (Table 6). No genetic linkage was observed among these loci. Fifteen loci with ‘*’ significantly departed from HWE after Bonferroni correction (*P*<0.05). Among the 26 SNP loci, three loci (S_Unigene508_c0_seq1_142, S_Unigene685_c0_seq1_3534, and S_Unigene508_c0_seq1_283) were related to epidermis formation, one locus (S_Unigene3026_c0_seq1_3726) was related to epidermis formation and muscle formation, three loci (S_Unigene512_c0_seq1_971, S_Unigene512_c0_seq1_5524, and S_Unigene512_c0_seq1_5912) were related to both vascularization and muscle formation, one locus (S_Unigene11849_c0_seq1_804) was related to the formation of blood vessels and skin, and the remaining loci were related to vascularization. Finally, we selected the *myosin heavy chain* gene, which was related to S_Unigene512_c0_seq1_971 among the 26 SNPs, for the detection of muscle development in the four species.

**Table 6:** Primer sequences and characterization of 26 SNPs in *Onchidium reevesii*.

Locus	PCR primers (F, R) and extension primer (P) sequences (5’–3’)	SNP	H_O_	H_E_	*P*	Function
*S_Unigene1402_c0_seq1_342	F:TGTCTGGCTATCCACTGA	G/A	0.5000	0.5000	0.0465	Hypothetical protein DAPPUDRAFT_228516
	S:TTCAGGATTCCTTTTGC					
	P:TTTTTTTTTTTTTTTTTTTTTTTAAGTGAGCATACCACATGCC					
*S_Unigene1402_c0_seq1_2685	F:TGTCCACTCCCAGCAGA	A/T	0.9818	0.0182	0.0000	Myosin VI
	S:GAGAATGCAGACAATACAAAA					
	P:TTTTTTTTTTTTTTTTTTTTTTTTTTTTTTTTGATGTAAAGTAAGCAGTGGAGC					
*S_Unigene1402_c0_seq1_471	F:TCCGAGGTTCCCTTGCT	A/T	1.0000	0.0000	0.0096	Myosin-VI-like
	S:GACAAAGAACAAGAAGAGGACA					
	P:TTTTTTTTTTTTTTTTTTTTTTTTTTTTTGACCAGCGAGCTCCTCATTC					
*S_Unigene508_c0_seq1_142	F:AGATGGACGCACCTTGT	T/A	0.9815	0.0185	0.0286	Ubc protein
	S:AAGTTTTCACAAAGATCTGCA					
	P:TTTTTTTTTTTTTTTTTTTAGTCTGAGCACCAAGTGGAG					
**S_Unigene1402_c0_seq1_1362**	F:TTGGCTTGAACTTGCGA	C/T	0.9825	0.0175	1.0000	Hypothetical protein DAPPUDRAFT_228516
	S:CAGTGGTGTACTCTGTCTGTGA					
	P:TTTTTTTTTTTTTTTTTTTTTTTTTTTTTTTTTTTTTTTTTTTTGTATCGTGCAGCCCA					
*S_Unigene1402_c0_seq1_1053	F:CCTGGAGTTTACGCAGT	C/A	1.0000	0.0000	0.0095	Myosin-VI
	S:CAAACATGGACGTCTTGA					
	P:TTTTTTTTATGACCAAGAGGCTGGCAGA					
*S_Unigene1402_c0_seq1_2178	F:TCCTTGTTGCGACTGTG	T/C	0.9032	0.0968	0.0000	Myosin-VI-like
	S:GTGGTATCTTTGACCTCCT					
	P:TTTTTTTTTTTTTTTTTTTTTTTTTTTTGGTGAAGTGATCATACTTTGG					
*S_Unigene1402_c0_seq1_99	F:GCCTACCCTTCCTCTACTT	T/A	0.4717	0.5283	0.0113	Myosin-VI-like
	S:TGGACCAGCACTACTCAA					
	P:TTTTTTTTTTTTTTTTTTTTAGGCCCAGAAAGTGGCTTC					
*S_Unigene685_c0_seq1_3534	F:GACCTCAAGGACCCACTG	C/T	0.9655	0.0345	0.0001	Col1a2
	S:CCTCAATAGGTTGGTCATACT					
	P:TTTTTTTTTTTTTTTTTTTTTTTTGCCCTTGCCAGCATAGTT					
**S_Unigene512_c0_seq1_971**	F:AATCCTATTCTGGAAGCCT	T/C	0.9455	0.0545	0.0889	Myosin heavy chain, non-muscle isoform X7
	S:AATCAAAGTTGATGCGG					
	P:TTTTTTTTTTTTTTTTTTTGCCAAGACCATCAAGAATGA					
**S_Unigene512_c0_seq1_5524**	F:GATGAACACACCAACACAGAG	A/T	0.9649	0.0351	0.9243	Myosin-10 isoform X6
	S:ACTGAGCGTTCAGAGGC					
	P:TTTTTTTTTTTTTTTTTTTTTTTTTTTTTTCATCTGCTCCACCTGGAGT					
*S_Unigene512_c0_seq1_591n2	F:GAGTGAAGGCCCTGAAA	G/A	0.5254	0.4746	0.0251	Myosin heavy chain
	S:TGCTCATCAAGTTCTCGC					
	P:TTTTTTTTTTTTTTTTTTTTTTTTTTTTTTTTTTTTTGGATGAGGCTGAGGAAGA					
*S_Unigene1402_c0_seq1_1359	F:TTGGCTTGAACTTGCGA	A/G	0.9583	0.0417	0.0050	LOC443649 protein, partial
	S:CAGTGGTGTACTCTGTCTGTGA					
	P:TTTTTTTTTTTTTTTTTTTTTTTTTTTTTTTTTTTTGTATCGTGCAGCCCAGTT					
*S_Unigene1402_c0_seq1_716	F:CTTGACGTGCGGCAACC	A/G	0.9556	0.0444	0.0006	Myosin-VI isoform 1
	S:CACAGGGACAGAGAACTGGC					
	P:TTTTTTTTTTTTTTTTTTTTTTTTTTTTTTTTTTACCAGGTGGAAGATATCACC					
**S_Unigene11849_c0_seq1_804**	F:CCAAGCCAAGAGGACTTA	G/A	0.4909	0.5091	0.5486	Mitogen-activated protein kinase
	S:CATGGGACTTTTGGTTT					
	P:TTTTTTTTTTTTTTTTTGGCAGGGACTGTATGTAACC					
**S_Unigene1402_c0_seq1_3336**	F:CAGCACTCTGTCAGGTACTT	T/C	0.9565	0.0435	0.0644	Hypothetical protein EGM_13779
	S:GTAACCAAGACCAGCCA					
	P:TTTTTTTTTTTTTTTTTTTTTTTTTTTTTTTTTTTTTTTGACTCGGTCTTCCCAGCTCC					
**S_Unigene3026_c0_seq1_3726**	F:AGGTCTAAGGTGGATGATTC	A/G	0.9825	0.0175	1.0000	Serine/arginine repetitive matrix protein 2-like
	S:TCTGGATTCTGAGGTGCT					
	P:TTTTTTTTTTTTTTAGATCTGAGCCAGAGGGCAG					
**S_Unigene1402_c0_seq1_300**	F:CAGCAACCATAAGAATAGGA	T/C	0.5893	0.4107	0.0976	Myosin-VI
	S:GCACGGCATGTGGTATG					
	P:TTTTTTTTTTTTTTTTTTTTTTTTTTTGATGGTCAGTGGATAGCCAG					
*S_Unigene1402_c0_seq1_1908	F:CAGTTGTTTCCTGAATTTG	T/G	1.0000	0.0000	0.0000	Myosin VI
	S:CGAAGAATCCATTGTTGA					
	P:TTTTTTTTTTTTTTTTTTTTTTTTTTTTTTTTTTTTTTGTTCCAGGATACAGAATCCAC					
**S_Unigene1402_c0_seq1_2823**	F:CCACCAGTGAATAGATACCTAA	A/T	0.9483	0.0517	0.0873	Jaguar, isoform I
	S:CGTGCTGGATGATGTCAA					
	P:TTTTTTTTTTTGCTGTGTGCGATGCAGC					
**S_Unigene394_c0_seq1_418**	F:ATGGATGGTACTGAAGGTCT	T/A	0.9000	0.1000	0.7108	H+ transporting ATP synthase beta subunit isoform 2
	S:ATGATTCTTCCGAGTGTCTT					
	P:TTTTTTTTGGTGAGCCCTGTGTGGACAT					
**S_Unigene1402_c0_seq1_1570**	F:CTCAGCAAACTTGCCCG	C/T	0.9211	0.0789	0.8371	CRE-SPE-15 protein
	S:CGATTGGATGCTAGGCTCT					
	P:TTTTTTTTTTTTTTTTTGGTCAAACCAAACTTAAAGTCC					
*S_Unigene508_c0_seq1_283	F:TTGAGCCATCTGACACAAT	T/C	0.9245	0.0755	0.0180	Ubc protein
	S:GCCATCCTCCAACTGTTT					
	P:TTTTTTTTTTTTTTTTTTTTTTTTTTTTTTTTTTTTTTTCAGGACAAAGAGGGAATCCC					
*S_Unigene1402_c0_seq1_2789	F:CAAAAGCAACATTGCCCA	A/T	0.9818	0.0182	0.0011	Protein SPE-15
	S:CGATGCAGCTATGAAGCAC					
	P:TTTTTTTTTTTTTTTTTTTTTTTTTTTTTTTTGCCACCAGTGAATAGATACCTA					
*S_Unigene1402_c0_seq1_2601	F:TTTCAGTGGCACCTTGAT	C/T	0.2553	0.7447	0.0000	AGAP000776-PA
	S:AAGGAGGAACTGAGGGA					
	P:TTTTTTTTCTGGTCAACCGTGTCATGCA					
**S_Unigene1402_c0_seq1_975**	F:CTTTTTCGCTCCAGCTCT	A/C	0.8000	0.2000	0.5133	Jaguar, isoform H
	S:GACTGCGTAAACTCCAGG					
	P:TTTTTTTTTTTTTTTTTTTTTTTTTTTTTTTTTTTTTTTGAGAAACAGCGCCGAGCAGA					

Observed heterozygosity (H_O_), expected heterozygosity (H_E_), significance of the test for deviation from HWE (*P*), and single nucleotide polymorphism (SNP).

### mRNA expression of *non-muscle myosin heavy chain II* in different tissues and different species

In mammals, NMHC II has three isoforms, which are referred to as NMHC IIA, NMHC IIB and NMHC IIC [28,29]. However, *Xenopus* has two isoforms, II-A and II-B, and does not appear to have an II-C isoform [30]. In invertebrates, *Drosophila* has only a single isoform of NH II [31,32]. According to the taxonomic placement of Onchidiidae, we speculated that Onchidium contained at least one isoform. The total length of the *Os-NMHC* sequence is 6057 bp. Furthermore, the specific expression of a gene in tissues is typically related to its function in those tissues, and different tissues reveal the different adaptions of a species. To investigate tissue-specific expression, the levels of mRNA expression in dorsal skin, ventral skin, lung sac, ganglion and ventricle samples from the four species were quantified by qRT-PCR. The SNPs reflected genetic differences among the four species of Onchidiidae. However, the expression differences of genes related to phenotype remained unknown. According to our histological study of Onchidiidae and the transcriptome data, we determined that *Os-NMHC* reveals the adaption from seas to wetlands in the four species of Onchidiidae. *Onchidium reevesii* shows apomorphic characters, as is evident from its ability to live in more complex environments [8]. We obtained the full-length cDNA, submitted it to the GenBank database and obtained an accession number (KU663401).

**Fig 5:**
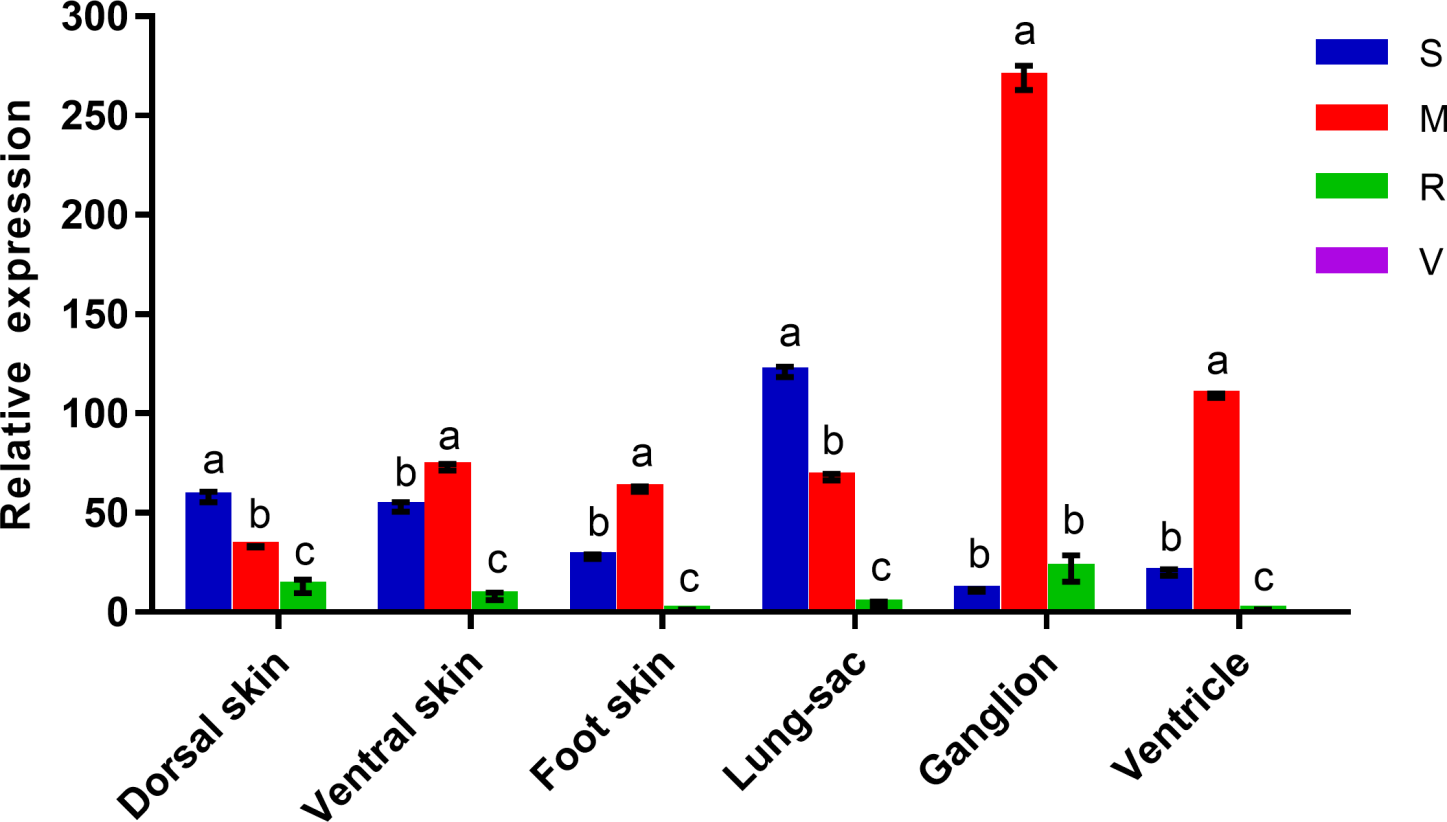
Expression levels of the *NMHC II* gene in different tissues from four representative Onchidiidae species. S = *Onchidium reevesii*; M = *Platevindex mortoni*; R = *Paraoncidium reevesii*; V = *Peronia verruculata* (little expression in the tested tissues).

The expression of *Os-NMHC* in various tissues of adults of the four species of Onchidiidae was determined (Fig 5). The *O*. *reevesii* has developed lung-sac observed among the four species; accordingly, this species presented high expression of the *Os-NMHC* gene in the lung-sac. In *Platevindex mortoni*, *Os-NMHC* exhibited the highest expression level in ganglion tissue, whereas *Os-NMHC* expression was not detected in most tissues of *Peronia verruculata*. The expression levels in the same tissue differed among the species (*P*<0.05).

### mRNA expression of *myosin heavy chain* in different tissues and different species

We cloned the *MyHC* gene (GenBank accession number: KU550708) of the four species and compared the expression levels in four types of tissues among the four species. The results showed that the full length of *MyHC* is 7566 bp. The expression level of *MyHC* was highest in *Onchidium reevesii* and lowest in *Paraoncidium reevesii*, and this difference was significant (*P*<0.05). The highest expression in the ventral skin and foot was observed in *Platevindex mortoni*. In the lung sacs, *P*. *mortoni* had the highest expression of *MyHC*, closely followed by *O*. *reevesii*, and *P*. *reevesii* showed the least expression.

**Fig 6:**
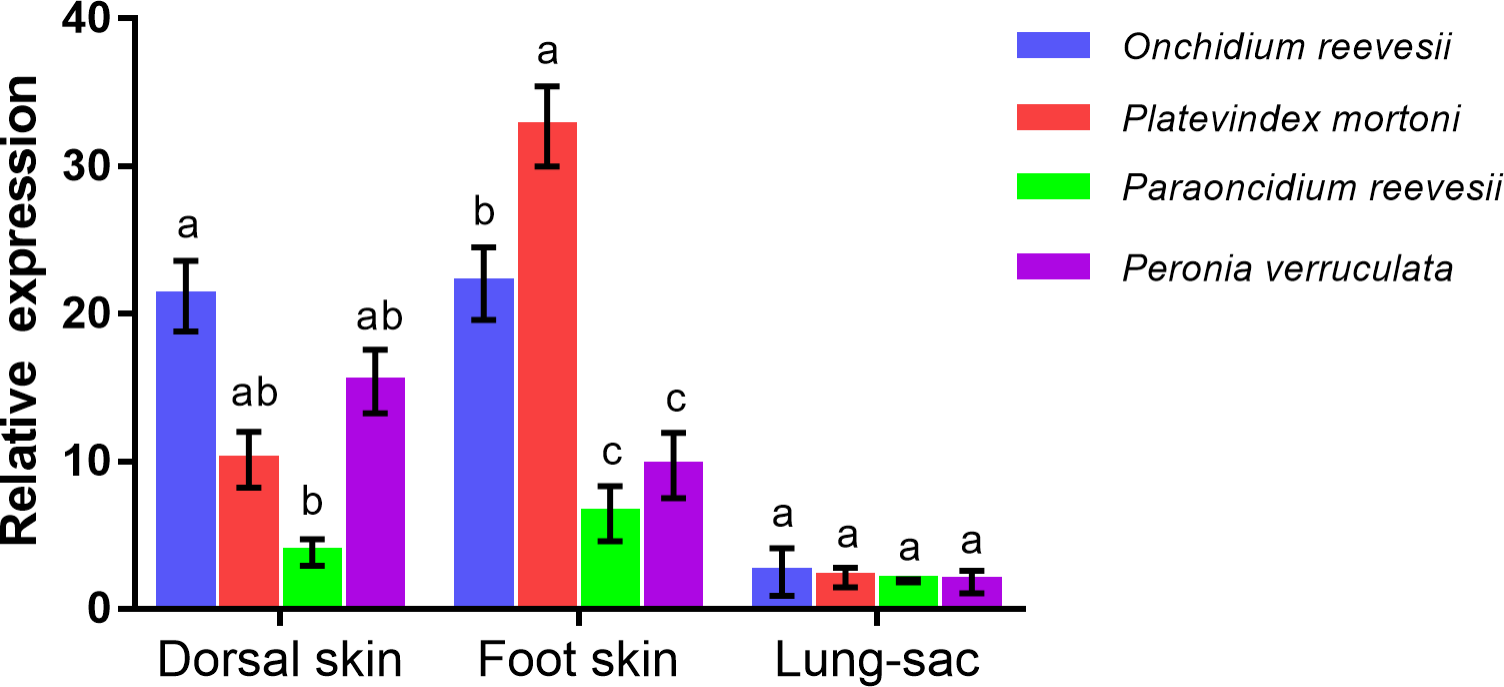
RT-qPCR analysis of the expression profiles of *MyHC* in different tissues of onchidiids.

We then analyzed the relative expression of the *MyHC* gene in three different tissues from the four Onchidiidae species to examine associations with living habitat (Fig 6). *O*. *reevesii* and *P*. *mortoni* are mainly terrestrial and burrow in mud or climb rocks to avoid the tide. However, *P*. *reevesii* and *P*. *verruculata* are mainly aquatic, and their movement requirements are lower. *O*. *reevesii* had high expression levels of *MyHC* in the dorsal skin, foot and lung sac, which are suited to their terrestrial habitat. *P*. *mortoni* expressed high levels in the foot, and this species frequently climbs trees. The epidermis of *P*. *verruculata* showed the highest level of keratinization observed among the four species; accordingly, this species presented high expression of the *MyHC* gene in the dorsal skin. We speculated that the expression level of this gene is related to the species' respiration ability, moisture retention ability and defense capacity.

## Discussion

Skin is an important respiratory organ for onchidiids, and skin with lower keratinization has higher permeability, which facilitates breathing in Onchidiidae species. In contrast, skin with higher keratinization helps retain moisture and protect against predators.

The skin of *Onchidium reevesii*, which is mainly terrestrial, has relatively weak respiratory function. The epidermis of this species is thick and functions in retaining moisture and protecting against predators, in accordance with its terrestrial characteristics [33,34]. The dorsal skin of the mainly aquatic *Paraoncidium reevesii* is thin and highly permeable; thus, its respiratory function is strong. Another aquatic species, *Peronia verruculata*, has a higher level of keratinization of the epidermis, but its gill skin is thin and suitable for breathing when submerged (Table 2). For *Platevindex mortoni*, skin thickness and the number of blood sinuses are intermediate among the four species. This species lives mostly in the supratidal zone and mudbank, can remain in the sea for long periods, and can climb trees.

The secretions from the mucous glands are slimy and smooth, which can reduce the friction between skin and water and facilitates gas exchange and ion transport [35]. Moreover, the dense distribution of blood sinuses is a hallmark trait of aquatic species [36]. There is only a small amount of blood sinuses in the stratum spongiosum of *Onchidium reevesii*, whereas the blood sinuses in the remaining species are abundant.

The sequence of the diameters of the sac room and the small room are also showed differences among the four species. Dayrat called the respiratory organ of *Onchidium vaigiense* a lung sac, which is similar to the breathing bag of limacines [37]. The developed lung sacs of amphibians are more suitable for terrestrial life [38]. The efficiency of lung sac respiration depends on the sac's superficial area. Thus, species with larger superficial areas have stronger respiratory capacities. Because of its well-developed reticular diaphragm, thin connective tissue, rich blood capillaries and largest superficial area of the lung sac among the four species, *O*. *reevesii* has the strongest respiratory capacity for wetland living among the four species. In conclusion, the degree of lung sac development in the four species of Onchidiidae decreased in the order *Onchidium reevesii*, *Peronia verruculata*, *Platevindex mortoni* and *Paraoncidium reevesii*.

The evolution of Onchidiidae species and their amphibious features is reflected in their morphological characteristics. Transcriptome sequencing data allow the study of genes related to the morphological differences and amphibious features of these typical amphibious mollusks. Our data provide the best transcriptomic resource currently available for these four species. The transcriptome data were obtained by Illumina HiSeq^TM^ 2000 sequencing, and the sequences were assembled and functionally annotated. Based on the annotated unigenes, GO and KEGG assignments were determined. This study established an excellent resource for future genetic or genomic studies of Onchidiidae variation and a platform for functional genomics and comparative genomic studies of mollusks.

In this study, SNP loci for *O*. *reevesii* were developed based on comparisons of its transcriptome sequences with those of the other three species. The different species adopted different strategies during their evolution. These loci of *O*. *reevesii* differed from those of *Peronia verruculata*, *Paraoncidium reevesii* and *Platevindex mortoni* and were related to vascularization and the formation of muscle and cuticle. These loci mutated readily and might reflect strong directional selection, which is important for understanding differences in respiratory traits and amphibious features among species. These loci might indicate reference genes and should be verified in other species. The SNPs might have undergone directional selection leading to adaptive evolution in Onchidiidae [39]. We selected the *myosin heavy chain* gene from among the SNP loci to investigate the different adaptations of the four species.

The dorsal skin plays significant roles in defending against predators and protecting against moisture loss. The developed ventral skin and foot skin are important for locomotion, and NMHC II can influence axon growth [40]. In some species, such as *Drosophila* [41], NMII is related to dorsal skin and is suggested to be involved in epidermal barrier functions [42]. Therefore, relevant to studies of epidermis adaption, NMHC II plays an important role in skin development. Our study revealed that the species that are mainly terrestrial (i.e., *Onchidium reevesii* and *Platevindex mortoni*) have a developed epidermis that can retain water and provide defense against predators. Their developed ventral skin and foot are suitable for rough terrain in the terrestrial environment. We found pronounced NMHC II expression in the skin (dorsal skin, ventral skin and foot skin) in the species that are mainly terrestrial, and we also found that *P*. *mortoni* showed high expression in the ventricle and ganglion. Previous studies found that the mutation of NMHC II affects the development of the heart [43,44]. Because the heart and ganglion are closely associated with feeding habits, this evidence explains how both *O*. *reevesii* and *P*. *mortoni* had the ability to adapt to terrestrial environments [45,46]. The observed higher-level expression of *Os-NMHC* in the respiratory and locomotive organs (dorsal skin, ventral skin, foot skin and lung sac) suggests that species that are mainly terrestrial (e.g., *O*. *reevesii* and *P*. *mortoni*) can easily adapt to complex land environments.

The myosin heavy chain protein is associated with muscle and is a suitable protein for analyzing muscle adaptation. Thus, its level of expression is associated with species habitat. The *myosin heavy chain* gene was thus selected from among the SNP loci for further examination. *O*. *reevesii* and *P*. *mortoni* mainly live in wetlands and must burrow in mud or climb rocks to avoid the tide. *P*. *reevesii* and *P*. *verruculata*, which are mainly aquatic, do not require strong terrestrial locomotor abilities. Moreover, the environment in which *P*. *reevesii* lives is more complex than the environments of the remaining species, and *P*. *reevesii* can climb trees. Therefore, *P*. *reevesii* has the strongest requirement for a developed foot, and its expression level of *MyHC* in its foot is much higher than the expression levels of this gene in the foots of *P*. *reevesii* and *P*. *verruculata*.

## Conclusion

Onchidiidae constitute an interesting group of invertebrates that occupy habitats ranging from seas to wetlands, showing a gradual distribution. Onchidiidae is an intermediary form that connects the invertebrates on land to those in the sea. In addition, this amphibious mollusk family offers a model for understanding histological and genetic changes associated with the water-to-land transition of invertebrates. Members of this group successfully made the transition from aquatic to terrestrial living and breathe with lung sacs, skin and gills to adapt to an amphibious lifestyle. Our histological analysis of respiratory organs from four onchidiids provides insights into their different adaptation. The 26 SNP loci that were selected can be used in further studies of respiratory traits and amphibious features. Moreover, we found that the relative expression levels of two genes are associated with the locomotor traits of the four species. We hope that this study provides a valuable reference point and a source of inspiration for future studies.

## References

[1] Gorkhali NA, Dong K, Min Y, Shen S, Kader A, Shrestha BS, et al (2016) Genomic analysis identified a potential novel molecular mechanism for high-altitude adaptation in sheep at the Himalayas. Scientific Reports 6: 29963 10.1038/srep29963 doi: 10.1038/srep29963 27444145 10.1038/srep29963 PMC4995607

[2] Qiang L, Fan S, Zhang Y, Meng X, Zhang H, Yang Y, et al (2016) The seahorse genome and the evolution of its specialized morphology. Nature 540: 395 10.1038/nature20595 doi: 10.1038/nature20595 27974754 10.1038/nature20595 PMC8127814

[3] Connie C. W. Hsia AS, Markus Lambertz, Steven F. Perry, John N. Maina (2013) Evolution of Air Breathing: Oxygen Homeostasis and the Transitions from Water to Land and Sky. Comprehensive Physiology 3: 849–915. 10.1002/cphy.c120003 doi: 10.1002/cphy.c120003 23720333 10.1002/cphy.c120003 PMC3926130

[4] Sagai T, Amano T, Maeno A, Kimura T, Nakamoto M, Takehana Y, et al (2017) Evolution of Shh endoderm enhancers during morphological transition from ventral lungs to dorsal gas bladder. Nature Communications 8: 14300 10.1038/ncomms14300 doi: 10.1038/ncomms14300 28155855 10.1038/ncomms14300 PMC5296767

[5] You X, Bian C, Zan Q, Xu X, Liu X, Chen J, et al (2014) Mudskipper genomes provide insights into the terrestrial adaptation of amphibious fishes. Nature Communications 5: 5594 10.1038/ncomms6594 doi: 10.1038/ncomms6594 25463417 10.1038/ncomms6594 PMC4268706

[6] Zardoya R, Meyer A (1996) The Complete Nucleotide Sequence of the Mitochondrial Genome of the Lungfish (Protopterus Dolloi) Supports Its Phylogenetic Position as a Close Relative of Land Vertebrates. Genetics 142: 1249–1263. 8846902 10.1093/genetics/142.4.1249 PMC1207122

[7] Bouchet P, Rocroi JP (2005) Classification and nomenclator of gastropod families. Malacologia 47: 1–368.

[8] Wu XF, Shen HD, Wu WJ, Zhang CJ, Wang L, Zhang Y (2010) Comparison on Morphology of Onchidiidae in Eastern Coast of China. Chinese Journal of Zoology 45: 92–100.

[9] Sun B, Chen C, Shen H, Zhang K, Zhou N, Qian J (2014) Species diversity of Onchidiidae (Eupulmonata: Heterobranchia) on the mainland of China based on molecular data. Molluscan Research 34: 62–70. 10.1080/13235818.2013.868860

[10] Wang D, Xu G, Qian J, Shen H, Zhang K, Guan J (2018) A morphological description of Onchidium reevesii (Gastropoda: Eupulmonata: Systellommatophora). Molluscan Research: 1–8. 10.1080/13235818.2018.1428780

[11] Ponder WF, Lindberg DR, Ponder WF, Lindberg DR (2008) Phylogeny and evolution of the mollusca: University of California Press 435–437 p.

[12] Pinchuck SC, Hodgson AN (2010) The ultrastructure and histology of the perinotal epidermis and defensive glands of two species of Onchidella (Gastropoda: Pulmonata). Tissue & Cell 42: 105–115. 10.1016/j.tice.2010.02.001 20206955 10.1016/j.tice.2010.02.001

[13] Huang XD, Zhao M, Liu WG, Guan YY, Shi Y, Wang Q, et al (2013) Gigabase-Scale Transcriptome Analysis on Four Species of Pearl Oysters. Marine Biotechnology 15: 253–264. 10.1007/s10126-012-9484-x doi: 10.1007/s10126-012-9484-x 23011005 10.1007/s10126-012-9484-x

[14] Talmadge RJ, Roy RR (1993) Electrophoretic separation of rat skeletal muscle myosin heavy-chain isoforms. Journal of Applied Physiology 75: 2337–2340. 10.1152/jappl.1993.75.5.2337 doi: 10.1152/jappl.1993.75.5.2337 8307894 10.1152/jappl.1993.75.5.2337

[15] Wessells NK, Spooner BS, Ash JF, Bradley MO, Luduena MA, Taylor EL, et al (1971) Microfilaments in cellular and developmental processes. Science 171: 135 10.1126/science.171.3967.135 5538822 10.1126/science.171.3967.135

[16] Franke JD, Montague RA, Kiehart DP (2005) Nonmuscle Myosin II Generates Forces that Transmit Tension and Drive Contraction in Multiple Tissues during Dorsal Closure. Current Biology 15: 2208–2221. 10.1016/j.cub.2005.11.064 doi: 10.1016/j.cub.2005.11.064 16360683 10.1016/j.cub.2005.11.064

[17] Bresnick AR (1999) Molecular mechanisms of nonmuscle myosin-II regulation. Current Opinion in Cell Biology 11: 26 10.1016/S0955-0674(99)80004-0 10047526 10.1016/s0955-0674(99)80004-0

[18] Pérez-Martínez C, García-Fernández RA, Ferreras-Estrada MC, Escudero-Diez A, Espinosa-Alvarez J, García-Iglesias MJ (1998) Optimization of the immunohistochemical demonstration of keratins in paraffin wax-embedded mouse skin. Journal of Comparative Pathology 119: 177–181. 10.1016/S0021-9975(98)80062-5 9749362 10.1016/s0021-9975(98)80062-5

[19] Grabherr MG, Haas BJ, Yassour M, Levin JZ, Thompson DA, Amit I, et al (2011) Full-length transcriptome assembly from RNA-Seq data without a reference genome. Nature Biotechnology 29: 644 10.1038/nbt.1883 doi: 10.1038/nbt.1883 21572440 10.1038/nbt.1883 PMC3571712

[20] Knowles DG, Mclysaght A (2009) Recent de novo origin of human protein-coding genes. Genome Research 19: 1752 10.1371/journal.pgen.1002379 doi: 10.1101/gr.095026.109 19726446 10.1101/gr.095026.109 PMC2765279

[21] Conesa A, Götz S, Garcíagómez JM, Terol J, Talón M, Robles M (2005) Blast2GO: a universal tool for annotation, visualization and analysis in functional genomics research. Bioinformatics 21: 3674–3676. 10.1093/bioinformatics/bti610 doi: 10.1093/bioinformatics/bti610 16081474 10.1093/bioinformatics/bti610

[22] Conesa A, Götz S (2008) Blast2GO: A Comprehensive Suite for Functional Analysis in Plant Genomics. International Journal of Plant Genomics 2008: 619832 10.1155/2008/619832 doi: 10.1155/2008/619832 18483572 10.1155/2008/619832 PMC2375974

[23] Götz S, Garcíagómez JM, Terol J, Williams TD, Nagaraj SH, Nueda MJ, et al (2008) High-throughput functional annotation and data mining with the Blast2GO suite. Nucleic Acids Research 36: 3420–3435. 10.1093/nar/gkn176 doi: 10.1093/nar/gkn176 18445632 10.1093/nar/gkn176 PMC2425479

[24] Ashburner M, Ball CA, Blake JA, Botstein D, Butler H, Cherry JM, et al (2000) Gene ontology: tool for the unification of biology. The Gene Ontology Consortium. Nature Genetics 25: 25–29. 10.1038/75556 doi: 10.1038/75556 10802651 10.1038/75556 PMC3037419

[25] Kanehisa M, Araki M, Goto S, Hattori M, Hirakawa M, Itoh M, et al (2008) KEGG for linking genomes to life and the environment. Nucleic Acids Research 36: 480–484. 10.1093/nar/gkm882 10.1093/nar/gkm882 PMC2238879 18077471

[26] Li H, Handsaker B, Wysoker A, Fennell T, Ruan J, Homer N, et al (2009) The Sequence Alignment/Map format and SAMtools. Bioinformatics 25: 2078–2079. 10.1093/bioinformatics/btp352 doi: 10.1093/bioinformatics/btp352 19505943 10.1093/bioinformatics/btp352 PMC2723002

[27] Sambrook J, Fritsch EF, Maniatis T (1989) Molecular cloning: a laboratory manual, 2nd ed. Analytical Biochemistry 186: 182–183.

[28] Conti MA, Adelstein RS (2008) Nonmuscle myosin II moves in new directions. Journal of Cell Science 121: 11 10.1242/jcs.007112 doi: 10.1242/jcs.007112 18096687 10.1242/jcs.007112

[29] Vicentemanzanares M, Ma X, Adelstein RS, Horwitz AR (2009) Non-muscle myosin II takes centre stage in cell adhesion and migration. Nature Reviews Molecular Cell Biology 10: 778 10.1038/nrm2786 doi: 10.1038/nrm2786 19851336 10.1038/nrm2786 PMC2834236

[30] Kelley CA, Sellers JR, Gard DL, Bui D, Adelstein RS, Baines IC (1996) Xenopus nonmuscle myosin heavy chain isoforms have different subcellular localizations and enzymatic activities. Journal of Cell Biology 134: 675–687. 10.1083/jcb.134.3.675 8707847 10.1083/jcb.134.3.675 PMC2120948

[31] Young PE, Richman AM, Ketchum AS, Kiehart DP (1993) Morphogenesis in Drosophila requires nonmuscle myosin heavy chain function. Genes & Development 7: 29. 10.1101/gad.7.1.29 8422986 10.1101/gad.7.1.29

[32] Peralta XG, Toyama Y, Hutson MS, Montague R, Venakides S, Kiehart DP, et al (2007) Upregulation of forces and morphogenic asymmetries in dorsal closure during Drosophila development. Biophysical Journal 92: 2583 10.1529/biophysj.106.094110 doi: 10.1529/biophysj.106.094110 17218455 10.1529/biophysj.106.094110 PMC1864829

[33] Bquay W (1972) Integument and the Environment Glandular Composition, Function, and Evolution. American Zoologist 12: 95–108. 10.1093/icb/12.1.95

[34] Arey LB, Barrick LE (1942) The structure of the repugnatorial glands of Onchidium floridanum. Journal of Morphology 71: 493–521. 10.1002/jmor.1050710305

[35] Phipps RJ, Torrealba PJ, Wanner A (1986) Development of ion transport and glycoprotein secretion in sheep trachea. Fed Proc, Fed Am Soc Exp Biol; (United States) 45:4: 1012–1012.

[36] Cao Y, Xie F, Jiang JP (2011) Histological Observation of Skin in Four Species in the Genus Scutiger. Sichuan Journal of Zoology 30: 214–211.

[37] Dayrat B (2010) Comparative anatomy and taxonomy of Onchidium vaigiense (Gastropoda: Pulmonata: Onchidiidae). Molluscan Diversity 2: 87–101.

[38] Hu CP, Shao Y, Ma QS (1998) Ultrastructural observation of different organs from Rana nigromaculata by scanning electron microscope(I). Journal of Xinxiang Medical College.

[39] Yoshiura K, Kinoshita A, Ishida T, Ninokata A, Ishikawa T, Kaname T, et al (2006) A SNP in the ABCC11 gene is the determinant of human earwax type. Nature Genetics 38: 324 10.1038/ng1733 doi: 10.1038/ng1733 16444273 10.1038/ng1733

[40] Hur EM, Yang IH, Kim DH, Byun J, Saijilafu Xu WL, et al (2011) Engineering neuronal growth cones to promote axon regeneration over inhibitory molecules. Proceedings of the National Academy of Sciences of the United States of America 108: 5057 10.1073/pnas.1011258108 doi: 10.1073/pnas.1011258108 21383151 10.1073/pnas.1011258108 PMC3064397

[41] Crish J, Conti MA, Sakai T, Adelstein RS, Egelhoff TT (2013) Keratin 5-Cre-driven excision of nonmuscle myosin IIA in early embryo trophectoderm leads to placenta defects and embryonic lethality. Developmental Biology 382: 136–148. 10.1016/j.ydbio.2013.07.017 doi: 10.1016/j.ydbio.2013.07.017 23911870 10.1016/j.ydbio.2013.07.017 PMC4186751

[42] Sumigray KD, Foote HP, Lechler T (2012) Noncentrosomal microtubules and type II myosins potentiate epidermal cell adhesion and barrier formation. Journal of Cell Biology 199: 513–525. 10.1083/jcb.201206143 doi: 10.1083/jcb.201206143 23091070 10.1083/jcb.201206143 PMC3483132

[43] Tullio AN, Accili D, Ferrans VJ, Yu ZX, Takeda K, Grinberg A, et al (1997) Nonmuscle myosin II-B is required for normal development of the mouse heart. Proceedings of the National Academy of Sciences of the United States of America 94: 12407–12412. 10.1073/pnas.94.23.12407 9356462 10.1073/pnas.94.23.12407 PMC24969

[44] Lu W, Seeholzer SH, Han M, Arnold AS, Serrano M, Garita B, et al (2008) Cellular nonmuscle myosins NMHC-IIA and NMHC-IIB and vertebrate heart looping. Developmental Dynamics 237: 3577–3590. 10.1002/dvdy.21645 doi: 10.1002/dvdy.21645 18697221 10.1002/dvdy.21645

[45] Grega DS, Prior DJ (1985) The effects of feeding on heart activity in the terrestrial slug, Limax maximus: central and peripheral control. Journal of Comparative Physiology A 156: 539–545. 10.1007/BF00613977

[46] Welsford IG, Prior DJ (1991) Modulation of heart activity in the terrestrial slug Limax maximus by the feeding motor program, small cardioactive peptides and stimulation of buccal neuron B1. Journal of Experimental Biology 155: 1–19. 2016573 10.1242/jeb.155.1.1

